# Prevalence, Comorbidities, and Risk Factors of Erectile Dysfunction: Results from a Prospective Real-World Study in the United Kingdom

**DOI:** 10.1155/2022/5229702

**Published:** 2022-03-20

**Authors:** Jim Z. Li, Terence A. Maguire, Kelly H. Zou, Lauren J. Lee, Shaantanu S. Donde, David G. Taylor

**Affiliations:** ^1^Viatris, 1000 Mylan Boulevard, Canonsburg, PA 15317, USA; ^2^School of Pharmacy, Queen's University Belfast, 97 Lisburn Rd, Belfast BT9 7BL, Belfast, UK; ^3^Patient and Health Impact, Pfizer Inc, 235 East 42nd St, NY, NY 10017, USA; ^4^Viatris, Trident Place Mosquito Way, Hatfield, Hertfordshire AL109UL, UK; ^5^School of Pharmacy, University College London, 29-39 Brunswick Square, London WC1N 1AX, UK

## Abstract

**Objectives:**

Assessment of erectile dysfunction (ED) burden could improve health outcomes associated with underlying cardiometabolic and psychological causes of ED. This study provided updated real-world evidence (RWE) on ED epidemiology and quantified healthcare resource utilization (HCRU) and health-related quality of life (HRQoL) burden among men with ED in the UK.

**Methods:**

This cross-sectional, prospective real-world evidence study was conducted via a self-reported Internet survey in 2018 in the UK general population. Prevalence of ED was estimated; HCRU and HRQoL were compared between men with ED versus without ED via bivariate analysis.

**Results:**

Of 12,490 men included, 41.5% reported ED; 7.5% of men reported severe ED; ED was most prevalent in Wales (44.3%). Men with ED were older (54.1 ± 14.5 vs. 46.8 ± 14.1 years) and often reported modifiable lifestyle risk factors, including smoking (32.8% vs. 26.3%), drinking alcohol (76.1% vs. 71.0%), not exercising (21.7% vs. 19.4%), and being overweight or obese (64.9% vs. 54.6%). Additionally, men with ED more often reported ≥1 comorbid chronic conditions (73.7% vs. 47.7%), including hypertension (31.8% vs. 16.3%), hyperlipidemia (27.6% vs. 14.0%), depression (24.3% vs. 14.6%), anxiety (23.3% vs. 16.6%), and diabetes (15.9% vs. 6.1%) versus men without ED (all, *p* < 0.001). Nearly half of men with ED (45.3%) were not undergoing treatment for cardiometabolic or psychological comorbidities. Furthermore, men with ED more often reported ≥1 visit to physicians/nurse practitioners and pharmacists in the past year and had significantly lower SEAR total and domain scores than men without ED (all, *p* < 0.001).

**Conclusion:**

ED was highly prevalent in the UK affecting over a quarter of younger men. Cardiometabolic and psychological conditions were common among men with ED and often remained untreated. Higher proportions of modifiable lifestyle risk factors observed among men with ED present an opportunity for healthcare providers to help mitigate the risk of cardiometabolic diseases and incidence of ED.

## 1. Introduction

Erectile dysfunction (ED), a consistent or recurrent inability to achieve and/or maintain an erection sufficient for sexual intercourse, has been estimated to affect up to three-quarters of men globally [1]. Furthermore, the prevalence of ED varies by geographic region [1, 2]. A prior retrospective epidemiological study among eight countries reported an overall 45.2% self-reported prevalence of ED in men aged 40–70 years, with Italy reporting the highest prevalence (52.2%) and Brazil reporting the lowest (42.1%) [2]. The wide variation in ED prevalence estimates across studies can be attributed to differences in the age groups examined and the manner in which ED was defined and assessed, among other methodological factors. Moreover, accurate estimation of the prevalence and burden of ED can be challenging, given ED is underdiagnosed and undertreated [3, 4].

The etiology of ED is multifactorial and is a complex interplay between vascular, neurological, hormonal, and psychological factors [5]. Additionally, vascular abnormalities of the penile blood supply and erectile tissue are closely associated with cardiovascular disease (CVD) and CVD risk factors [6]. In a systematic review, Raheem and colleagues (2017) noted that ED is a sentinel marker of subclinical CVD and vascular dysfunction [7]. In a prior meta-analysis, ED was identified as an independent predictor of cardiovascular events [8]. The incidence and severity of ED increase with advancing age and the presence of underlying comorbidities, such as CVD and diabetes [5]. Previous epidemiological research has reported that among men with ED, approximately 40% have hypertension; 42% have hyperlipidemia; and 20% have diabetes [2]. Due to the close connection between cardiometabolic diseases and ED, current guidelines of the British Society for Sexual Medicine recommend that men initially presenting with ED should be thoroughly screened and treated, when appropriate, for cardiometabolic risk factors [6]. In particular, the screening of younger men presenting with ED symptoms may help identify those who are at high risk of developing CVD [7, 9]. Ensuring such screenings are performed by HCPs as part of routine medical care will facilitate cardiometabolic risk mitigation and the earlier diagnosis and management of cardiometabolic diseases.

ED has been associated with work productivity loss and poor mental and physical health-related quality of life (HRQoL), relative to men without ED [10, 11], although it is likely these relationships can be at least partially explained by underlying cardiometabolic and psychological conditions. Furthermore, the partners of men with ED often report experiencing relationship difficulties, less frequent sexual activity, and decreased sexual satisfaction [10]. Thus, the burden associated with ED can negatively affect men, as well as their partners.

Prior research has often focused on middle-aged and elderly men with ED [2], whereas the prevalence and burden of ED in the broader adult male population have received somewhat less attention. The inclusion of younger men with ED in research studies has important implications, as evaluation for CVD risk factors can facilitate the earlier identification of men who are at high risk for cardiovascular events [9]. As the most recent epidemiological data from the UK are ≥5 years old [2], elucidating the current epidemiology and burden of ED in the UK is warranted. A refreshed understanding of ED prevalence and burden will aid healthcare providers (HCPs) in improving the detection and management of ED and its fundamental cardiometabolic and psychological causes. Accordingly, this prospective study aimed to provide updated real-world evidence (RWE) on ED epidemiology, as well as quantify healthcare resource utilization (HCRU) and HRQoL burden among men with ED in the UK general population.

## 2. Materials and Methods

### 2.1. Study Design and Sample

This cross-sectional, prospective real-world evidence (RWE) study was conducted via a self-reported Internet survey between March and April 2018. Adult men (aged ≥18 years) who provided electronic informed consent and were able to read and write in UK English were included in the study. Participants were recruited through the opt-in online panel of Kantar Profiles (formerly, Lightspeed Research) that represents the demographic characteristics of the general adult population in the UK. Respondents were recruited from diverse online sources, such as partner panels and opt-in e-mail invitations. All panelists agreed to be panel members and completed an in-depth demographic registration profile. Potentially eligible panelists were contacted via e-mail to participate in the study. Panelists receive “points” for completing a survey, which are deposited in their panel account and can later be redeemed for products, online gift certificates, or a cash honorarium. All panel members undergo a double-opt-in process, including a registration process followed by activation via e-mail. Only respondents who have activated their accounts are included in the panel. The study protocol was reviewed and granted an exemption by the Sterling Institutional Review Board (Atlanta, GA, USA).

### 2.2. Study Measures

#### 2.2.1. ED Status and Severity

ED status and severity were assessed based on a single-item measure of self-reported erection problems from the Massachusetts Male Aging Study. This measure has been shown to accurately predict ED diagnosis via urological examination and distinguish between men who have ED and those who do not have ED [12]. Men who reported experiencing a mild, moderate, or severe erection problem in the past month were categorized as having ED. Those who reported no erection problem in the past month were categorized as not having ED.

#### 2.2.2. Sociodemographic and General Health Characteristics

Data collected on sociodemographic characteristics included age, race, education, annual income, current employment status, marital status, current relationship status (if not married/living with a domestic partner), current relationship duration (if married/living with a domestic partner or in a relationship, but not living with a domestic partner), and geographic region in the UK. General health characteristics assessed included height and weight measurements used to calculate body mass index (BMI). Additionally, data on being sexually active, sexual intercourse frequency per month, overall health status, overall level of life stress, smoking, alcohol consumption, and exercise frequency in the past week were also collected.

#### 2.2.3. Comorbidities

Data on underlying comorbidities diagnosed by an HCP, including cardiometabolic and psychological conditions, were collected. Additionally, depression was assessed via the Patient Health Questionnaire-2 (PHQ-2), a two-item measure that has been validated as a depression screening tool [13, 14]. The PHQ-2 evaluates the frequency in the past two weeks of the two core Diagnostic and Statistical Manual of Mental Disorders, Fourth Edition (DSM-IV) clinical criteria for a diagnosis of major depressive disorder (i.e., anhedonia and depressed mood). Scores on the PHQ-2 can range from 0 to 6; scores ≥3 indicate a positive screen for major depressive disorder. Men also reported on whether they currently used any of the following prescription treatments: alpha blockers (for a heart condition), antidepressants, blood pressure medication, statins, diabetes medication, nitrates, or vasodilators.

#### 2.2.4. Healthcare Resource Utilization

HCRU was assessed based on the number of physician/nurse practitioner and pharmacist visits in the past year. Specifically, the number of physician/nurse practitioner visits for any reason, including visits to cardiologists and for CVD check-ups, and the number of pharmacist visits for any reason, including visits for sexual functioning discussions, were assessed. Further details on the items used to assess HCRU in this study have been reported elsewhere [15].

#### 2.2.5. Health-Related Quality of Life

HRQoL was measured via the Self-Esteem and Relationship Questionnaire (SEAR) [16]. This 14-item measure evaluates the impact of ED on psychosocial functioning and well-being. The questionnaire measures sexual relationship satisfaction, overall relationship satisfaction, confidence, and self-esteem in men with ED. Total scores, as well as scores on each domain, range from 0 to 100, with higher scores indicating better HRQoL. A difference of ≥10 points in total or domain scores is considered a minimal clinically important difference [17] (MCID).

#### 2.2.6. Statistical Methods

All statistical analyses were performed using IBM SPSS^®^ version 23.0 or higher. Period prevalence of ED was estimated among the total study sample and for the subsets of men aged 18–39 and ≥40 years. The period prevalence of mild, moderate, and severe ED among the total study sample was also estimated. Additionally, the period prevalence of ED was estimated by UK region (England, Scotland, Wales, or Northern Ireland). Prevalence estimates were reported as percentages with two-sided 95% confidence intervals (CIs).

Descriptive statistics for all study variables were reported for the total study sample and separately for men with ED and men without ED. Means and standard deviations (SDs) were reported for continuous and discrete variables; medians and interquartile ranges (IQRs) were additionally reported for skewed study variables. Frequencies and percentages were reported for categorical variables. Men with and without ED were compared on all study variables (sociodemographic and general health characteristics, underlying comorbidities, HCRU, and HRQoL) using independent-samples *t*-tests (or Mann–Whitney *U* tests, for skewed variables) for continuous or discrete variables and chi-square tests for categorical variables. Two-sided *p* values <0.05 were considered statistically significant.

## 3. Results

A total of 12,506 adult men participated in the survey from the UK general population. Of these, a total of 16 participants were excluded from the analysis due to multiple quality issues (e.g., straight-lining (i.e., entering the identical response option for all items in a series of questions that use the same response scale), illogical responses, and extreme outliers) resulting in a final study sample of 12,490 participants.

### 3.1. Prevalence of ED

Among the total study sample, 5,185 men had ED based on the single-item self-report measure, which corresponded to an overall estimated prevalence of 41.5% (95% CI: 40.7%–42.4%). The prevalence of mild, moderate, and severe ED in the total study sample was estimated at 20.2% (95% CI: 19.5%–20.9%), 13.8% (95% CI: 13.3%–14.5%), and 7.5% (95% CI: 7.1%–8.0%), respectively. Among men aged 18–39 years (*n* = 3,118), 29.3% (95% CI: 27.7%–30.9%) had ED, whereas the prevalence of ED among men aged ≥40 years (*n* = 9,372) was 45.6% (95% CI: 44.6%–46.6%). ED was most prevalent among men who reside in Wales (*n* = 626; 44.3% (95% CI: 40.4%–48.2%)) and least prevalent among men who reside in Northern Ireland (*n* = 234; 40.2% (95% CI: 34.1%–46.6%)). Among men who reside in Scotland (*n* = 1,095) and England (*n* = 10,522), the prevalence of ED was estimated at 43.6% (95% CI: 40.7%–46.5%) and 41.2% (95% CI: 40.2%–42.1%), respectively.

### 3.2. Sociodemographic and General Health Characteristics

Relative to men without ED, men with ED were older (54.1 ± 14.5 vs. 46.8 ± 14.1 years) and more often reported having an annual income <£50,000 (74.9% vs. 66.7%) and being married/living with a domestic partner (69.2% vs. 62.1%; all, *p* < 0.001; Table 1). Additionally, men with ED were less likely to be currently employed than men without ED (55.7% vs. 69.9%; *p* < 0.001). Statistically significant differences between men with ED and those without ED were also observed in the distribution for the UK region of residence (*p* < 0.001). Overall, most men with ED (83.5%) and men without ED (84.8%) resided in England; men with ED more often resided in Scotland or Wales than men without ED (14.5% vs. 13.3%).

Men with ED were in poorer health than men without ED, engaged in less sexual activity, and more frequently reported modifiable lifestyle risk factors associated with ED (Table 2). Specifically, men with ED more often reported smoking (32.8% vs. 26.3%), drinking alcohol (76.1% vs. 71.0%), and not exercising (21.7% vs. 19.4%) in the past week than men without ED. Relative to men without ED, those with ED more often had overweight or obese BMI (64.9% vs. 54.6%); additionally, men with ED less frequently rated their overall health status as very good or excellent (29.6% vs. 45.0%) and more frequently rated their overall level of life stress as extremely or very stressful (19.0% vs. 15.7%), relative to men without ED (all, *p* < 0.001).

Men with ED less frequently reported being sexually active than men without ED (66.3% vs. 69.1%; *p* < 0.001; Table 2). Similarly, men with ED also reported having had sexual intercourse fewer times in the past month than men without ED (median (IQR): 3.0 (0.0, 6.0) vs. 4.0 (1.0, 8.0); *p* < 0.001).

### 3.3. Comorbidities

Men with ED more often reported being diagnosed by an HCP with ≥1 comorbid chronic condition, relative to men without ED (73.7% vs. 47.7%; *p* < 0.001; Table 3). All individual comorbidities assessed were more common among men with ED than men without ED, including cardiometabolic and psychological conditions. Particularly, men with ED more frequently reported being diagnosed with hypertension (31.8% vs. 16.3%), high cholesterol (27.6% vs. 14.0%), anxiety (23.3% vs. 16.6%), and diabetes (15.9% vs. 6.1%; all, *p* < 0.001). Men with ED also screened positive for depression on the PHQ-2 more often than men without ED (24.3% vs. 14.6%; *p* < 0.001). Among men with ED, 54.7% reported currently using ≥1 of the following treatments for underlying cardiometabolic or psychological conditions: alpha blockers (for a heart condition), antidepressants, blood pressure medication, statins, diabetes medication, nitrates, or vasodilators, nearly double that for men without ED (28.0%; Table 4).

### 3.4. Healthcare Resource Utilization

Men with ED reported significantly higher HCRU than men without ED in the past 12 months on all types of HCP visits assessed (all, *p* < 0.001; Table 5). Those with ED reported ≥1 visits to see cardiologists (7.5% vs. 2.8%), have CVD check-ups (17.9% vs. 8.5%), and see pharmacists for sexual functioning discussions (14.9% vs. 3.9%) approximately two to three times more frequently than men without ED (all, *p* < 0.001).

### 3.5. Health-Related Quality of Life

**Figure 1:**
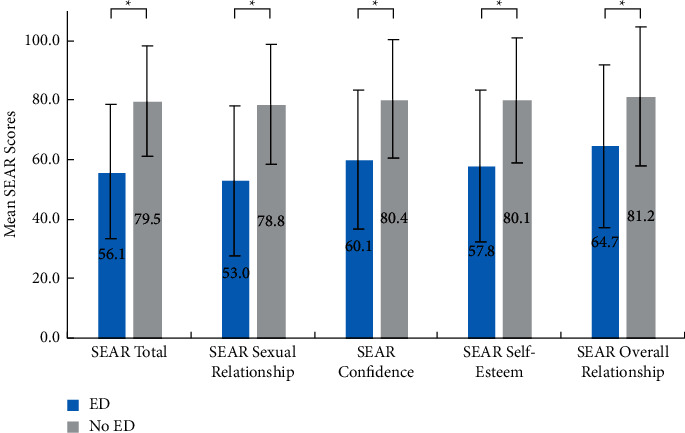
Health-related quality of life in men with ED and men without ED error bars that represent SD values. ED = erectile dysfunction; SD = standard deviation; SEAR = Self-Esteem and Relationship Questionnaire. ^∗^*p* < 0.001, two-sided.

HRQoL was lower in men with ED than in men without ED, with the differences exceeding the MCID of 10 points for all SEAR metrics **(**Figure 1). Men with ED had significantly lower mean SEAR total (56.1 vs. 79.5) and domain scores, including sexual relationship (53.0 vs. 78.8), confidence (60.1 vs. 80.4), self-esteem (57.8 vs. 80.1), and overall relationship (64.7 vs. 81.2; all, *p* < 0.001).

## 4. Discussion

This prospective study provides real-world evidence (RWE) on the prevalence of self-reported ED in the UK general adult population. Results of this survey demonstrated a high prevalence of ED, with over two in five men aged ≥18 years affected. The prevalence rate observed in the current study is consistent with a previous estimate of 42.6% for the UK based on data from the 2015 and 2016 National Health and Wellness Surveys and similar to the ED prevalence rates reported for other developed countries, including 48.6% in Italy, 44.9% in France, 44.9% in Germany, 43.5% in Spain, and 42.0% in the USA [2]. Results also revealed moderate variations by the UK region, with Wales and Scotland having higher ED prevalence than England and Northern Ireland, suggesting a greater need may exist in the former two regions for HCP intervention to diagnose and treat chronic cardiometabolic conditions and support healthy lifestyle changes.

The link between advancing age and higher ED prevalence is well-established [1]. Likewise, in the UK population, we observed an approximately 1.6-fold higher prevalence among those aged ≥40 years than those <40 years. Notably, nearly three in ten men aged <40 years in this study self-reported having ED, indicating that the burden of ED is not isolated to middle-aged and older age groups, a disturbing trend that has also been documented in a handful of previous studies [1, 9, 18]. However, due to erroneous beliefs that ED is a self-limiting condition among younger men, their ED symptoms may be disregarded without further medical assessment [9]. Given the cardiometabolic and psychological conditions known to underlie ED [19], the high ED prevalence rate observed among men aged <40 years in the current study warrants further attention by HCPs and supports the need for better detection and management of the fundamental causes of ED, especially among younger men.

In the current study, modifiable lifestyle risk factors, including overweight/obese BMI, drinking alcohol, smoking, and physical inactivity, were significantly more common among men who self-reported ED than men who self-reported no ED. In a recent meta-analysis of 20 cohort studies involving 1,090,261 participants, healthier lifestyle behaviors were associated with a significantly lower risk of cardiovascular events during the average 12.3-year follow-up period, although that study did not specifically focus on ED [20]. However, taking into account the results reported by Tsai and colleagues (2020) and evidence demonstrating that ED is an independent indicator of cardiovascular event risk [8], it can be inferred that engaging in more salutary lifestyle behaviors that improve cardiovascular health can, in turn, reduce the incidence of ED. As lifestyle risk factors are largely modifiable, they represent a key area for HCPs to target interventions that provide men with appropriate consultation and support to limit their alcohol intake, quit smoking, increase exercise frequency, and achieve and maintain optimal body weight.

Men with self-reported ED in the current study reported significantly higher rates of cardiometabolic and psychological comorbidities, including hypertension, high cholesterol, depression, anxiety, and diabetes, than men without ED. Yet nearly half of men with self-reported ED in this study were not currently using any pharmacotherapy for these conditions, which suggests unmet needs in the detection and management of the cardiometabolic and psychological conditions, potential causes of ED. However, medication use, including certain classes of antihypertensives and antidepressants, can also potentially cause ED symptoms [21], which may at least partially explain the higher rates of medication use observed among men with self-reported ED, relative to men without ED, in this study. If medication use is suspected as the cause of an individual's ED, physicians can consider substituting the current medication with an alternative that is less likely to impair sexual functioning, if deemed medically appropriate for that individual [21].

In the present study, HCRU was found to be greater in the past year in men with self-reported ED than those without ED, in terms of the number of visits to physicians/nurse practitioners for any reason, cardiologist visits, and visits for CVD check-ups, as well as visits to pharmacists for any reason and for sexual functioning discussions. A recent UK-based study reported high HCRU (i.e., physician/nurse and pharmacist visits) in the 12 months following baseline assessment in men with ED, with HCRU being even higher among men who used non-prescription sildenafil from the pharmacy [15]. Thus, taken together with the results from the current study, men with ED may have greater engagement with the healthcare system than men without ED, and having readily accessible ED treatment may serve to further increase contact with HCPs among men with ED. Of importance, the greater healthcare system engagement of men with ED may potentially provide HCPs with additional opportunities to monitor and assess cardiometabolic risk factors and manage chronic cardiometabolic diseases among these individuals. Ideally, all HCPs, including community pharmacists, will also engage more actively in supporting salutary lifestyle changes.

In the current study, men with self-reported ED reported significantly lower SEAR scores than men without ED, indicating worse HRQoL due to ED. As differences between men with self-reported ED and men without ED exceeded the 10-point MCID threshold for all SEAR metrics, these results suggest that the negative influence of ED on HRQoL may be clinically meaningful. The SEAR scores observed in our study are consistent with those reported by men with ED from the UK in a prior cross-sectional study conducted across eight countries [22]. Other research has also reported sizable differences between men with ED and men without ED using alternative validated measures of HRQoL, such as summary scores and health utilities from the Short-Form 12-Item Health Survey [11]. While prior research indicates that using a phosphodiesterase type 5 inhibitor to treat ED is associated with better HRQoL, as measured via SEAR [15,16], the earlier diagnosis and management of underlying cardiometabolic and psychological conditions, as well as changing unhealthy lifestyle behaviors, can improve both health status and HRQoL outcomes [23]. Hence, improving HRQoL among men with ED will likely require HCPs to use a multipronged approach that simultaneously addresses ED symptoms and the health conditions and risk factors that fundamentally cause and exacerbate these symptoms.

The current study was conducted prior to the COVID-19 pandemic; although in light of emerging evidence showing an increase in unhealthy lifestyle behaviors during the pandemic, it is possible that this study's results provide a conservative estimate of the current burden of ED in the UK. There have been several publications on the associations between COVID-19 and ED. For example, Sansone et al. (2020) reviewed and summarized the research on the pathophysiological mechanisms associating COVID-19 to ED [24]. The authors reported the increased prevalence of ED among COVID-19 patients [25] and discussed how long-term complications of COVID-19 could adversely affect sexual function. Therefore, it might be used as a biomarker for the severity and complications of COVID-19 [26]. In addition, Goldstein et al. conducted a real-world data study and reported that COVID-19 significantly decreased the number of ED patients in the USA receiving ED-related treatments [27]. For instance, social isolation during pandemic lockdown was associated with increased alcohol consumption, decreased physical activity levels, and increased sedentary behavior [28, 29]. Thus, the impact of COVID-19 further underscores the need to address lifestyle factors, such as smoking, lack of exercise, and obesity, that put individuals at greater risk for CVD, as well as for COVID-19 infection.

### 4.1. Limitations

The results of our study might be subject to recall bias, particularly for HCRU outcomes that had a 12-month recall period. Given the use of convenience sampling, results may also have been affected by selection bias, such that healthier individuals were more likely to participate than men with more severe comorbidities or disabilities, although it was not possible to determine if men who chose to participate in the study differed in any systematic way from men who opted not to participate. However, to help minimize potential selection bias and increase generalizability, sampling quotas based on the age group to mimic UK men's age distribution were implemented to ensure greater representativeness of the UK adult male population.

All data in this study were self-reported. Thus, diagnoses, treatment, and HCRU could not be independently verified. However, ED status and depression were assessed using well-established, validated self-report measures. As the study data are cross-sectional, causality cannot be inferred from the results, and temporal fluctuations in the prevalence of ED and underlying cardiometabolic and psychological conditions could not be evaluated.

## 5. Conclusion

This study provides real-world data on the prevalence of self-reported ED and its causal risk factors in the UK general population. ED prevalence in the UK general population was high, overall, and affected over a quarter of younger men. Cardiometabolic and psychological comorbid chronic conditions were common among men with self-reported ED, although many were not receiving pharmacological treatment. As ED is a marker for cardiometabolic diseases, increasing awareness among men and encouraging them to seek medical consultation earlier, when ED symptoms are initially noticed, will be vital for improving detection and management of these underlying conditions. Higher proportions of modifiable lifestyle risk factors observed among men with self-reported ED further present an opportunity for HCPs to intervene to help mitigate cardiometabolic disease risk and reduce the incidence of ED.

## Figures and Tables

**Table 1:** Sociodemographic characteristics of men with ED and men without ED.

Variable	ED status		Two-sided *p* value
	ED (*n* = 5,185)	No ED (*n* = 7,305)	
Age, mean (SD)	54.11 (14.49)	46.83 (14.11)	<0.001
Age group, *n* (%)			<0.001
18–39	914 (17.6)	2,204 (30.2)	
40–49	1,019 (19.7)	2,104 (28.8)	
50–64	1,672 (32.2)	2,077 (28.4)	
≥65	1,580 (30.5)	920 (12.6)	
Race, *n* (%)			<0.001
White	4,777 (92.1)	6,604 (90.4)	
Non-white	370 (7.1)	602 (8.2)	
Prefer not to answer	38 (0.7)	99 (1.4)	
Education, *n* (%)			0.024
No university degree	2,548 (49.1)	3,541 (48.5)	
University degree	2,608 (50.3)	3,691 (50.5)	
Prefer not to answer	29 (0.6)	73 (1.0)	
Income, *n* (%)			<0.001
<£50,000	3,882 (74.9)	4,871 (66.7)	
≥£50,000	1,070 (20.6)	1,860 (25.5)	
Prefer not to answer	233 (4.5)	574 (7.9)	
Employment, *n* (%)			<0.001
Unemployed	2,273 (43.8)	2,121 (29.0)	
Employed	2,889 (55.7)	5,108 (69.9)	
Prefer not to answer	23 (0.4)	76 (1.0)	
Marital status, *n* (%)			<0.001
Married/domestic partner	3,588 (69.2)	4,539 (62.1)	
Not married	1,597 (30.8)	2,766 (37.9)	
Current relationship status, *n* (%)^†^			<0.001
In relationship, not living with partner	425 (26.6)	643 (23.2)	
Not in relationship, have ≥1 partner	266 (16.7)	312 (11.3)	
Not in relationship, do not have partner	906 (56.7)	1,811 (65.5)	
Current relationship duration, *n* (%)^§^			<0.001
<1 year	125 (3.1)	243 (4.7)	
1 to <3 years	246 (6.1)	358 (6.9)	
3 to <5 years	250 (6.2)	430 (8.3)	
5 to <10 years	476 (11.9)	756 (14.6)	
10 to <20 years	788 (19.6)	1,318 (25.4)	
≥20 years	2,128 (53.0)	2,077 (40.1)	
Region, *n* (%)			<0.001
Scotland	477 (9.2)	618 (8.5)	
Wales	277 (5.3)	349 (4.8)	
Northern Ireland	94 (1.8)	140 (1.9)	
England	4,330 (83.5)	6,192 (84.8)	
Other UK regions	7 (0.1)	6 (0.1)	

Numbers for some variables may not be added to the total sample due to missing data. ED, erectile dysfunction; SD, standard deviation; UK, United Kingdom. ^†^The item on current relationship status was only asked of the subset of men who reported that they were not married/living with a domestic partner. ^§^The item on current relationship duration was only asked of the subset of men who reported that they were married/living with a domestic partner or in a relationship, but not living with a domestic partner.

**Table 2:** General health characteristics of men with ED and men without ED.

Variable	ED status		Two-sided *p* value
	ED (*n* = 5,185)	No ED (*n* = 7,305)	
BMI, mean (SD)	27.91 (5.76)	26.91 (5.51)	<0.001
BMI category, *n* (%)			<0.001
Underweight	76 (1.6)	125 (1.9)	
Normal weight	1,382 (28.6)	2,401 (36.8)	
Overweight	2,009 (41.6)	2,544 (39.0)	
Obese	1,359 (28.2)	1,447 (22.2)	
Sexually active, *n* (%)			<0.001
Yes	3,438 (66.3)	5,050 (69.1)	
No	1,617 (31.2)	1,915 (26.2)	
Prefer not to answer	130 (2.5)	340 (4.7)	
Number of times have sexual intercourse per month, mean (SD)	4.83 (6.67)	6.03 (8.40)	<0.001^∗^
Median (IQR)	3.00 (0.00, 6.00)	4.00 (1.00, 8.00)	
Overall health status, *n* (%)			<0.001
Excellent	283 (5.5)	891 (12.2)	
Very good	1,247 (24.1)	2,398 (32.8)	
Good	1,828 (35.3)	2,494 (34.1)	
Fair	1,300 (25.1)	1,191 (16.3)	
Poor	527 (10.2)	331 (4.5)	
Overall level of life stress, *n* (%)			<0.001
Extremely stressful	286 (5.5)	308 (4.2)	
Very stressful	701 (13.5)	839 (11.5)	
Moderately stressful	1,662 (32.1)	2,588 (35.4)	
Somewhat stressful	1,373 (26.5)	1,942 (26.6)	
Not at all stressful	1,163 (22.4)	1,628 (22.3)	
Smoking-past week, *n* (%)			<0.001
0 days	1,950 (37.6)	2,491 (34.1)	
1–2 days	301 (5.8)	257 (3.5)	
3–4 days	257 (5.0)	204 (2.8)	
5–7 days	1,141 (22.0)	1,458 (20.0)	
Never smoker	1,536 (29.6)	2,895 (39.6)	
Alcohol consumption-past week, *n* (%)			<0.001
0 days	1,041 (20.1)	1,638 (22.4)	
1–2 days	1,930 (37.2)	2,960 (40.5)	
3–4 days	1,065 (20.5)	1,295 (17.7)	
5–7 days	956 (18.4)	937 (12.8)	
Never drinks alcohol	193 (3.7)	475 (6.5)	
Exercise-past week, *n* (%)			<0.001
0 days	1,125 (21.7)	1,414 (19.4)	
1–2 days	1,647 (31.8)	2,177 (29.8)	
3–4 days	1,314 (25.3)	1,858 (25.4)	
5–7 days	1,099 (21.2)	1,856 (25.4)	

Numbers for some variables may not be added to the total sample due to missing data. BMI, body mass index; ED, erectile dysfunction; IQR, interquartile range; SD, standard deviation. ^∗^The two-sided *p* value shown was generated via a Mann–Whitney *U* test.

**Table 3:** Diagnosed comorbidities reported in men with ED and men without ED.

Comorbidities	ED status		Two-sided *p* value
	ED (*n* = 5,185)	No ED (*n* = 7,305)	
AIDS/HIV, *n* (%)	79 (1.5)	31 (0.4)	<0.001
Anxiety, *n* (%)	1,208 (23.3)	1,209 (16.6)	<0.001
Any cancer (not prostate), *n* (%)	300 (5.8)	179 (2.5)	<0.001
Metastatic solid tumor (not prostate), *n* (%)	46 (0.9)	12 (0.2)	<0.001
Prostate cancer, *n* (%)	175 (3.4)	47 (0.6)	<0.001
Chronic pulmonary disease, *n* (%)	243 (4.7)	121 (1.7)	<0.001
Congestive heart failure, *n* (%)	70 (1.4)	23 (0.3)	<0.001
Heart attack, *n* (%)	252 (4.9)	118 (1.6)	<0.001
Diabetes-chronic complications, *n* (%)	81 (1.6)	21 (0.3)	<0.001
Diabetes-no chronic complications, *n* (%)	744 (14.3)	426 (5.8)	<0.001
Dementia, *n* (%)	34 (0.7)	7 (0.1)	<0.001
Enlarged prostate, *n* (%)	466 (9.0)	184 (2.5)	<0.001
Heart disease, *n* (%)	265 (5.1)	119 (1.6)	<0.001
Depression^†^, *n* (%)	1,258 (24.3)	1,066 (14.6)	<0.001
Hemiplegia/paraplegia, *n* (%)	27 (0.5)	11 (0.2)	<0.001
Hypertension, *n* (%)	1,649 (31.8)	1,192 (16.3)	<0.001
High cholesterol, *n* (%)	1,433 (27.6)	1,022 (14.0)	<0.001
Kidney disease, *n* (%)	93 (1.8)	60 (0.8)	<0.001
Liver disease (mild), *n* (%)	92 (1.8)	49 (0.7)	<0.001
Liver disease (moderate to severe), *n* (%)	33 (0.6)	24 (0.3)	0.012
Rheumatologic disease, *n* (%)	314 (6.1)	245 (3.4)	<0.001
None of the above, *n* (%)	1,365 (26.3)	3,818 (52.3)	<0.001

AIDS/HIV, acquired immunodeficiency syndrome/human immunodeficiency virus; ED, erectile dysfunction. ^†^Positive depression screen (i.e., score ≥3 on Patient Health Questionnaire-2).

**Table 4:** Pharmacological treatments for underlying cardiometabolic and psychological comorbidities in men with ED and men without ED.

Medications	ED status		Two-sided *p* value
	ED (*n* = 5,185)	No ED (*n* = 7,305)	
Alpha blocker-heart conditions, *n* (%)	300 (5.8)	108 (1.5)	<0.001
Antidepressants, *n* (%)	891 (17.2)	719 (9.8)	<0.001
Blood pressure medication, *n* (%)	1,677 (32.3)	1,034 (14.2)	<0.001
Statins, *n* (%)	1,479 (28.5)	845 (11.6)	<0.001
Diabetes medication, *n* (%)	712 (13.7)	355 (4.9)	<0.001
Nitrates, *n* (%)	170 (3.3)	46 (0.6)	<0.001
Vasodilators, *n* (%)	114 (2.2)	28 (0.4)	<0.001
≥1 of the above, *n* (%)	2,836 (54.7)	2,047 (28.0)	<0.001

ED, erectile dysfunction.

**Table 5:** Healthcare resource utilization in men with ED and men without ED in the past 12 months.

Visits to HCPs	ED status		Two-sided *p* value
	ED (*n* = 5,185)	No ED (*n* = 7,305)	
Physician/nurse practitioner visits for any reason, *n* (%)			<0.001
No visits	1,083 (20.9)	2,921 (40.0)	
1 or more visits	4,094 (79.0)	4,377 (59.9)	
Visits to cardiologist, *n* (%)^†^			<0.001
No visits	3,794 (92.5)	4,259 (97.2)	
1 or more visits	307 (7.5)	121 (2.8)	
Physician/nurse visits for CVD check-up, *n* (%)^†^			<0.001
No visits	3,364 (82.1)	4,009 (91.5)	
1 or more visits	733 (17.9)	371 (8.5)	
Pharmacist visits for any reason, *n* (%)			<0.001
No visits	3,074 (59.3)	5,002 (68.5)	
1 or more visits	2,111 (40.7)	2,303 (31.5)	
Pharmacist visits for sexual functioning discussions, *n* (%)^§^			<0.001
No visits	1,794 (85.1)	2,211 (96.1)	
1 or more visits	314 (14.9)	90 (3.9)	

Numbers for some variables may not be added to the total sample due to missing data. CVD, cardiovascular disease; ED, erectile dysfunction; HCP, healthcare provider. ^†^The items on visits to cardiologists and for CVD check-ups were only asked of the subset of men who reported ≥1 visit to a physician/nurse practitioner for any reason. ^§^The item on visits for sexual functioning discussions was only asked of the subset of men who reported ≥1 visit to a pharmacist for any reason.

## Data Availability

Data supporting the findings of this study may be available for noncommercial use from the corresponding author upon reasonable request.
